# *Mycoplasma* infection and ocular surface diseases: a nationwide cohort study

**DOI:** 10.1038/s41598-021-01941-9

**Published:** 2021-11-22

**Authors:** Li-Ju Lai, Vincent Chin-Hung Chen, Yao-Hsu Yang, Kai-Liang Kao, Ko-Jung Chen, Ying-Ching Wang, Shu-I Wu

**Affiliations:** 1 University Eye Center, Chia-Yi, Taiwan, ROC; 2 Department of Psychiatry/Health Information and Epidemiology Laboratory, Chang Gung Memorial Hospital, Chia-Yi, Taiwan, ROC; 3 Department of Psychiatry, Chang Gung University, Tao-Yuan city, Taiwan, ROC; 4 Department of Traditional Chinese Medicine, Chiayi Chang Gung Memorial Hospital, Chia-Yi, Taiwan, ROC; 5 School of Traditional Chinese Medicine, College of Medicine, Chang Gung University, Tao-Yuan City, Taiwan, ROC; 6 Health Information and Epidemiology Laboratory, Chang Gung Memorial Hospital, Chia-Yi, Taiwan, ROC; 7 Department of Pediatrics, Far Eastern Memorial Hospital, New Taipei City, Taiwan, ROC; 8 Department of Ophthalmology, Taipei City Hospital, Renai Branch, Taipei, Taiwan, ROC; 9 Department of Medicine, Mackay Medical College, New Taipei City, Taiwan, ROC; 10 Department of Psychiatry, Mackay Memorial Hospital, Taipei, Taiwan, ROC

**Keywords:** Eye diseases, Epidemiology, Clinical microbiology, Infectious-disease diagnostics

## Abstract

Whether patients with *Mycoplasma* infection have an increased risk of ocular surface ulcers. Using a nation-wide database, we identified patients with a new diagnosis of *Mycoplasma* infection between 1997 and 2013, and compared them with age-, sex-, and index year-matched subjects without the infection. Cox proportional regression was performed to compare the risk of corneal diseases between the two cohorts. The incidence of corneal diseases was significantly higher in the 4223 patients with *Mycoplasma* infection than in the 16,892 patients without (7.28 vs. 5.94 per 1000 person-years, *P* < 0.01). The adjusted hazard ratio for the risk of corneal diseases in the study cohort was 1.21 times higher (95% CI 1.02–1.44) than that in the comparison cohort. *Mycoplasma* infection might be a predisposing factor for patients with keratitis.

## Introduction

Microbial corneal ulcer is a common but potentially sight-threatening ocular infection caused by infectious microorganisms or noninfectious stimuli^1^. Common pathogens include bacterial agents, such as *Staphylococcus aureus*, *Pseudomonas aeruginosa*, and *Streptococcus pneumonia*; fungal agents, such as *Fusarium* and *Aspergillus*; and protists. Clinical history taking, detailed clinical examination, or a corneal tissue culture can facilitate pathogen identification^2^. However, the rate of successful laboratory cultivation for microbial corneal ulcer in humans has varied widely from 22 to 75%^3–5^. The low rates of culture positivity may be due to the lack of corneal scrape samples, the inhibition of microbial growth by antibiotic treatment before corneal tissue culture, the use of inappropriate growth medium, mixed or atypical clinical features, or the coexistence of other pathogens^3,6^.

*Mycoplasma* is a recognized pathogen that colonizes the mucosal surfaces of humans and animals^7^. *Mycoplasma pneumoniae* may cause upper and lower respiratory tract infections in all age groups and is responsible for up to 40% of community-acquired atypical pneumonia^8^. Extrapulmonary manifestations have been reported in various systems, including the cardiovascular, neurological, dermatological, hematological, musculoskeletal, gastrointestinal, and urogenital systems^7,9,10^. A history of *Mycoplasma* pulmonary infection or positive findings of serum antibodies are helpful for diagnosing these extrapulmonary infections. Several human case reports of ocular infections caused by *Mycoplasma*, such as anterior uveitis^11–13^ and papillitis^9,14,15^, have been published. For ocular surface infections, although *Mycoplasma* is a primary pathogen causing severe infectious keratoconjunctivitis in animals^16–18^, its role as a pathogen in human corneal ulcer has rarely been described. Only few cases of *Mycoplasma* conjunctivitis have been reported^19,20^. The low rate of detection of *Mycoplasma* as an etiology for human corneal ulcer can be attributed to the difficulty in obtaining a high rate of culture positivity^8^. Sanchez-Vargas et al. reported that the sensitivity of culture for *Mycoplasma* in extrapulmonary samples was less than 60%^10^. Qu et al. reported that only 55.6% of patients with *Mycoplasma pneumoniae* showed positive culture results^21^. In addition to direct damage to host cells, previous literature discussed that *Mycoplasma*-related conjunctivitis or uveitis may be attributed to systemic inflammation or immunological reactions to *Mycoplasma*^7,22,23^. Studies investigating the association between human ocular surface diseases and *Mycoplasma* infection are lacking. By using a large, nationwide, population-based database and clinical experience, we conducted a longitudinal cohort study in Taiwan to evaluate whether patients with *Mycoplasma* infection may have an increased risk of corneal infection.

## Methods

### Data source

We used Taiwan’s National Health Insurance (NHI) Research Database (NHIRD) to identify and enroll our study and comparison patients. Taiwan launched its single-payer compulsory NHI program in 1995, and this insurance program currently covers 99% of the population in Taiwan. The NHIRD has been released for research purposes and contains claims data on diagnoses, drug prescriptions, and intervention receipts, but not laboratory results, obtained from ambulatory care, emergency services, and hospitalizations^24^. All personal details on the enrollees of the NHI are encrypted^24^. Diagnoses are coded according to the International Classification of Diseases, Ninth Revision, Clinical Modification (ICD-9-CM) criteria. From the NHIRD, we identified patients who had received a new diagnosis of *Mycoplasma pneumonia* (*M. pneumonia*) infection (ICD-9-CM 483.0) (those with first hospitalization and a discharge diagnosis of *M. pneumoniae* infection) between 1997 and 2013. The index date was the date of first hospitalization for *M. pneumoniae* infection. For each study patient, 4 age-, sex-, index date-, urbanization-, and income-matched comparison patients without *M. pneumoniae* infection were selected. We only included hospitalized patients with a discharge diagnosis of *M. pneumoniae* infection was because most patients with *M. pneumoniae* infection do not need hospitalization and could be treated in outpatient services. If we include patients from outpatient services, there might be an underestimation for *M. pneumoniae* infection and an over-estimation for non-infection because not all patients had symptoms or signs serious enough to receive clinical attention. While comparing the hospitalized patients with and without *M. pneumoniae* infection, we were able to minimize misclassifications of case and comparison groups. Comorbidities were defined as the diagnosis of blepharitis (ICD-9-CM code 373) or glaucoma (ICD-9-CM code 365) before the index date. The Charlson Comorbidity Index (CCI) was calculated and compared between the study and comparison patients. Patients with a history of corneal diseases (ICD-9-CM codes 370 and 371) before the index date or those with incomplete age or sex information were excluded.

### Main outcome

The main outcome of this study was the date of corneal infection or diseases (ICD-9-CM code 370.xx [keratitis] and 371.xx [corneal opacity and other disorders of the cornea]). All study and comparison patients were followed from the index date until the date of corneal disease diagnoses, withdrawal from the insurance system (i.e. death, jailed, served in the army, or foreigners that exceeded the permitted stay or working permits), loss to follow-up, or the end of the follow-up (31 December 2013), whichever occurred earlier.

### Statistical analysis

We used the χ^2^ test to determine differences in categorical variables and comorbidities before the index date between patients with and without *M. pneumoniae* infection. Student’s *t* test was used to examine the difference in the mean age between the two groups. Incidence rates (per 1000 person-years) for corneal or ocular surface infections stratified by having *M. pneumoniae* infection, demographic variables, or comorbidities were calculated. The Kaplan–Meier method was used to measure the cumulative incidence, and the log-rank test was used to assess the differences in incidence rates between the 2 groups. Univariable and multivariable Cox proportional hazard regression analyses were performed to estimate and compare crude and adjusted hazard ratios (HRs) and 95% confidence intervals (CIs) for subsequent corneal diseases between study and comparison patients. All statistical analyses were performed using SAS software (version 9.4, SAS Institute, Cary, NC, USA). The Kaplan–Meier analysis was performed using R software (R Foundation for Statistical Computing, Vienna, Austria). A *P* value of < 0.05 was considered statistically significant. All study protocols were approved by the Institutional Review Board of Chang Gung Memorial Hospital (approval no: 201600959B0).

## Results

We included 4223 patients with *M. pneumoniae* infection in the study cohort and 16,892 age-, sex-, index year-, and sociodemographic-matched patients without *M. pneumoniae* infection in the comparison cohort. Both the study and comparison cohorts showed no significant difference in the distribution of age, sex, urbanization level, or income level (Table 1). The study cohort had significantly higher CCI scores (*P* < 0.0001) and higher blepharitis infection rates (*P* = 0.001) before the index date than did the comparison cohort. The mean latency period between the initial *M. pneumoniae* infection and the diagnosis of corneal infections or diseases was 5.3 years.

**Table 1:** Demographic characteristics of and comorbidities in patients with and without *Mycoplasma pneumonia* enrolled in Taiwan’s National Health Insurance Research Database.

Variables	Mycoplasma (N = 4223)		Non-mycoplasma (N = 16,892)		p value
	n	%	n	%	
**Gender**					1.000
Male	1988	47.08	7952	47.08	
Female	2235	52.92	8940	52.92	
**Age (year)**					1.000
< 18	2804	66.40	11,216	66.40	
18–45	779	18.45	3116	18.45	
> 45	640	15.16	2560	15.16	
**Urbanization level**					1.000
1 (City)	1112	26.33	4448	26.33	
2	1945	46.06	7780	46.06	
3	801	18.97	3204	18.97	
4(Villages)	365	8.64	1460	8.64	
**Income**					1.000
0	3231	76.51	12,924	76.51	
1 ~ 15,840	255	6.04	1020	6.04	
15,841 ~ 25,000	439	10.40	1756	10.40	
> 25,000	298	7.06	1192	7.06	
**Charlson Comorbidity Index (CCI)**					< 0.001
≦2	4055	96.02	16,597	98.25	
> 2	168	3.98	295	1.75	
**Comorbidities**					
Blepharitis					0.001
Yes	673	15.94	2352	13.92	
No	3550	84.06	14,540	86.08	
**Glaucoma**					0.055
Yes	75	1.78	233	1.38	
No	4148	98.22	16,659	98.62	

The incidence of corneal infections or diseases was significantly higher in the study cohort than in the comparison cohort (7.28 vs. 5.94, respectively, per 1000 person-years), with an adjusted HR of 1.21 (95% CI 1.02–1.44, *P* = 0.03; Table 2). The risk of corneal ulcer or corneal diseases was significantly higher in female and older patients, those who lived in the most urbanized areas, those who had higher CCI scores, and those who had received a diagnosis of blepharitis before the index date.

**Table 2:** Cox model with the hazard ratio (HR) and 95% confidence interval (CI) of corneal and ocular surface infection with potential covariates.

Variables	Crude				Adjusted^a^			
	HR	95% CI		p value	HR	95% CI		p value
Mycoplasma (yes/no)	1.23	1.03	1.46	0.023	1.21	1.02	1.44	0.033
Gender (male/female)	0.78	0.67	0.91	0.001	0.80	0.68	0.93	0.003
**Age (year)**								
< 18	1.00	Reference			1.00	Reference		
18–45	1.00	0.80	1.26	0.980	1.02	0.77	1.36	0.872
> 45	1.70	1.38	2.09	< 0.001	1.69	1.26	2.26	< 0.001
**Urbanization level**								
1(City)	1.72	1.25	2.35	0.001	1.68	1.22	2.31	0.001
2	1.25	0.91	1.70	0.167	1.24	0.91	1.69	0.184
3	1.12	0.79	1.57	0.535	1.09	0.77	1.53	0.633
4(Villages)	1.00	Reference			1.00	Reference		
**Income**								
0	1.00	Reference			1.00	Reference		
1 ~ 15,840	0.93	0.65	1.35	0.710	0.79	0.53	1.18	0.246
15,841 ~ 25,000	1.25	0.97	1.61	0.087	0.96	0.69	1.33	0.794
> 25,000	1.34	0.99	1.82	0.056	1.05	0.72	1.52	0.806
**Charlson Comorbidity Index (CCI)**								
≦2	1.00	Reference			1.00	Reference		
> 2	2.14	1.36	3.38	0.001	1.49	0.91	2.42	0.112
**Comorbidity (yes/no)**								
Blepharitis	1.57	1.31	1.88	< 0.001	1.56	1.30	1.87	< 0.001
Glaucoma	2.01	1.24	3.26	0.004	1.47	0.90	2.41	0.124

Crude HR: relative hazard ratio; Adjusted HR: adjusted hazard ration, mutually adjusted for age, sex, levels of income and urbanizations, Charlson Comorbidity Index scores, and comorbidities of blepharitis and glaucoma.

**Figure 1:**
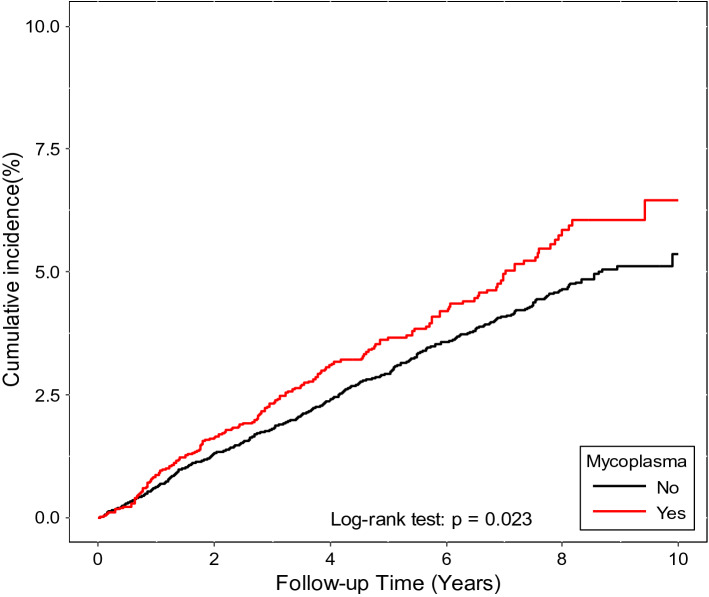
Cumulative incidence of corneal infection in patients with and without *Mycoplasma pneumonia* infection.

Figure 1 shows Kaplan–Meier curves for the cumulative incidence of corneal ulcers or diseases between the study and comparison cohorts. A significantly higher risk of corneal diseases was found in the study cohort than in the comparison cohort (log-rank test, *P* = 0.012).

## Discussion

In the present cohort study, we investigated the association between *M. pneumoniae* infection and subsequent corneal ulcers or diseases. To the best of our knowledge, this is the first population-based longitudinal cohort study to examine such association. After adjusting for sex, age group, income level, urbanization level, CCI score, and comorbidities, the patients with *M. pneumoniae* infection exhibited a 1.21-fold higher risk of ocular surface diseases, including corneal ulcer, ocular phemphegoid, or cornea degeneration, than those without *M. pneumoniae* infection. Such result suggested increased associations between having the history of Mycoplasma infection and subsequent corneal diseases. *Mycoplasma* may be considered as a precursor for ocular surface predisposition to keratitis, although it is usually neglected because of the difficulty in obtaining culture or DNA confirmations.

Although a strong association between *Mycoplasma* and infectious keratoconjunctivitis in animals has been described, few human cases of *Mycoplasma* conjunctivitis have been reported in the literature^19,20^. No previous cohort study has examined the association between human systemic *M. pneumoniae* infection and subsequent ocular surface involvements. Although the pathomechanisms through which *M. pneumoniae* causes a wide range of extrapulmonary infections remain largely unknown, the following 3 nonmutually exclusive mechanisms are proposed^22^. (1) Direct *Mycoplasma* invasion: Since the cornea has no blood vessels, the pathogen of *M. pneumoniae* may be transmitted through tears or aqueous fluid diffusions to infiltrate the cornea. Such process takes longer time than the hematogenous transmissions. In addition, Mycoplasmal antigens, such as glycoproteins and glycolipids, may activate local inflammatory cytokines (eg, interleukin [IL]-6 and IL-8) to induce inflammatory responses. (2) Indirect immune modulation: the absence of *Mycoplasma* infection at the site of inflammation, whereas the manifestation in distant organs may be caused through autoimmunity or immune complex formation^22^. (3) Vascular occlusion type: extrapulmonary or neurological manifestations of *Mycoplasma* infection may be caused by the obstruction of blood flow associated with the formation of thrombosis, embolism, vasculitis, or vasculopathy involving an immune complex (eg, antiphospholipid antibodies)^7,22,23^. However, it remained unclear whether ocular surface involvements in our study patients were due to the direct spread of *M. pneumoniae* to the eyes or a *Mycoplasma*-induced immune response cross-reacting with the ocular surface tissue. Our finding that the mean latency period between the initial *Mycoplasma* infection and subsequent corneal disease diagnosis was not immediate but an average of 5 years later suggests possible chronic influences of vascular occlusions involving immune complexes. These observations might be consistent with the aforementioned mechanisms, resulting in complicated clinical manifestations.

The prevalence of microbiology varied in different geographical regions. Research showed that in China, prevalence of viral (43.6%) or bacterial (46.2%) keratitis accounted for nearly 90% of the corneal infection^25^. Whereas in Uganda, over half of the keratitis were fungal (62%), 7% were mixed bacterial and fungal, 7% bacterial, and 24% no organism detected^26^. Often times, the pathogen identification for corneal ulcer requires history taking, detailed clinical examinations, or the facilitation of corneal tissue culture^2^. However, the rate of successful laboratory cultivation for microbial corneal ulcer in humans has varied widely from 22 to 75%^3–5^. In bacterial infections, rapid inflammation, dense stromal suppuration, and ground glass appearance on cornea are usually observed. In fungal keratitis, stromal infiltrates with filament edges, satellite lesions, and thick endothelial exudate are often described. The ring-shaped infiltration could also be seen in fungal, herpes simplex, or amoeba keratitis^30^. *Mycoplasma* infection presented quite differently from typical infections caused by the pathogens mentioned above. Macrolides (eg. azithromycin and clarithromycin) are the treatment of choice for *Mycoplasma* infection. It can not only eradicate the microorganism but also reduce the amount of potentially harmful bacterial components, such as the bacterial cell wall^7^. Furthermore, macrolides may be beneficial in managing the indirect-type extrapulmonary manifestations of *Mycoplasma* infection by suppressing the immune responses of the host^7,27,28^. In addition to antibacterial activity, macrolides have immunomodulatory properties than can enhance host defense mechanisms in the first few weeks of drug initiation. Prolonged curtailment of local inflammation or neutrophil activities also helps resolving infection or inflammation^29^. *Mycoplasma* is may be an overlooked predisposing factor of keratitis, even when the examining tools were not good enough, the ophthalmologist's high degree of clinical suspicion on different manifestations of co-infections and relevant treatments may help give patients a better treatment result.

The strengths of this study include the use of a nationwide database to conduct large longitudinal cohorts with adequate statistical power to detect the association of interest. There are some limitations in this study. First, the results of laboratory tests, culture findings, and imaging studies were not available in the NHIRD; and significant differences were found on underlying comorbidities and blepharitis between the two cohorts. It is also possible that the higher rates of ocular surface disease might be correlated with the underlying comorbidities. Although we have tried to adjust these differences and our result still revealed a significantly higher risk of corneal ulcer in the Mycoplasma infection group within the stratification of people with blepharitis (adjusted HR 1.57, 95% CI 1.30 ~ 1.87, Table 2), we still lack sufficient information to explore possible causal mechanisms in this longitudinal observational study. Future clinical studies with sufficient laboratory assessments are still required. Second, patients with mild *Mycoplasma* infection might not have been hospitalized for treatment. In accordance with our inclusion criteria, we identified study patients who had *Mycoplasma* infection sufficiently severe to require hospitalization for clinical management. Hence, our findings might only be generalized to the relatively severely affected subgroups of people. Third, information for the longitudinal cohort was drawn from datasets developed for administrative rather than for research purposes. Diagnoses obtained from these datasets reflect real-world clinical practice and might not necessarily generalize for researching diagnostic criteria. Under- or over- estimations for diagnoses of Mycoplasma infection and corneal diseases may occur. However, previous literature has described moderate to substantial agreements between the NHIRD claim data and medical charts (Kappa values of 0.55 ~ 0.86). In addition, all insurance claims (including diagnoses and managements given) were scrutinized regular by peer supervision. If fraud is identified, penalties would be given to physicians. Hence, the diagnoses in our study may be judged as reliable. Fourth, no information on lifetime *Mycoplasma* infection before the launch of the NHI system could be obtained, and we could conduct analyses using only information obtained during our follow-up window (i.e. 1997 to 2013). Such measurement errors occurred in both exposure and outcome; therefore, the association of interest might have been attenuated rather than exaggerated.

## Conclusion

The results of our population-based observational study revealed an increased association between *Mycoplasma* infection and subsequent corneal diseases. *Mycoplasma* infection may be considered as a precursor for ocular surface predisposition to keratitis. Appropriate laboratory examinations, as well as adequate therapeutic managements, may be applied if the possibility of *Mycoplasma* infection is suspected. Additional large prospective noninterventional studies are required to elucidate the microbiological, immunological, and hematological causal mechanisms^22^.
